# COVID-19 and Lung Cancer Interactions: A Literature Review

**DOI:** 10.3390/medsci13040295

**Published:** 2025-11-30

**Authors:** Szabolcs-Attila László, Edith-Simona Ianoși, Anca-Meda Văsieșiu, Mioara Szathmáry, Maria Beatrice Ianoși, Delia-Liana Rachiș, Gabriel Nistor, Gabriela Jimborean

**Affiliations:** 1 Doctoral School of Medicine and Pharmacy, University of Medicine, Pharmacy, Science and Technology “George Emil Palade” of Târgu Mureș, 540142 Târgu Mureș, Romania; szaby1994@gmail.com (S.-A.L.); gabriela.jimborean@umfst.ro (G.J.); 2 Department of Pulmonology, University of Medicine and Pharmacy, Science and Technology “George Emil Palade” of Târgu Mureș, 540139 Târgu Mureș, Romania; mioara.szathmary@umfst.ro; 3 Department of Infectious Disease, University of Medicine, Pharmacy, Science and Technology “George Emil Palade” of Târgu Mureș, 540139 Târgu Mureș, Romania; anca-meda.vasiesiu@umfst.ro; 4 Infectious Diseases Clinic 1, Mureș Clinical County Hospital Mures, 540233 Târgu Mureș, Romania; 5 Pulmonology Clinic, Mureș County Clinical Hospital, 540011 Târgu Mureș, Romania; ianosi.maria-beatrice@stud18.umfst.ro (M.B.I.); deliarachis@yahoo.ro (D.-L.R.); 6 Aivita Biomedical Inc., Irvine, CA 92612, USA; gabriel@aivitabiomedical.com

**Keywords:** SARS-CoV-2, COVID-19, lung cancer, long COVID, diagnostic deficit, healthcare disruption, tumor microenvironment, cytokines and chemokines, autophagy and inflammasomes, stage migration

## Abstract

This review aims to discuss the apparent reduction in pulmonary cancer incidence in the general population during and shortly after the COVID-19 pandemic from a biological and pathophysiological mechanistic point of view. While the epidemiological evidence points to a disruption in the early- and mid-stage diagnostic process, which causes a shift to late-stage lung cancer discovery with no impact on its actual prevalence, an alternative hypothesis based on the intersection of viral and cancer biology could have a real effect on lung carcinogenesis as an independent phenomenon. By weaving together population-level trends, mechanistic insights, and translational oncology, we discuss whether the pandemic-associated decline in lung cancer diagnoses reflects primarily a temporary diagnostic artifact or whether it also reveals biologically relevant intersections between SARS-CoV-2 and pulmonary oncogenesis. The COVID-19 pandemic, caused by the severe acute respiratory syndrome coronavirus 2 (SARS-CoV-2), has exerted profound and multifaceted effects on global healthcare systems, altering patterns of disease detection, management, and outcomes across nearly all medical disciplines. These disruptions generated what has been termed a “diagnostic deficit”, producing a backlog of undetected cancers that have only partially been recovered in subsequent years. This phenomenon, sometimes described as a “COVID-19 debt” in oncology, is thought to contribute to excess late-stage diagnoses and potentially worse medium-term survival outcomes. Beyond the disruption of medical systems, the pandemic also raised a more speculative but biologically intriguing question: could SARS-CoV-2 infection itself, through direct or indirect mechanisms, influence lung cancer biology? Our review aims to critically synthesize the evidence across seven domains to address this dual hypothesis. (1) We examine the observed effects of the pandemic on cancer incidence, highlighting global registry and health-system data; (2) we review SARS-CoV-2 infection biology, including viral entry, replication, protein functions, and treatment implications; (3) we summarize the pathogenesis of lung cancer; (4) we explore the role of immune checkpoints in tumor immune evasion, followed by (5) analyses of immune dysregulation in acute infection and (6) in long COVID; and (7) finally, we evaluate proposed oncogenic mechanisms of SARS-CoV-2, integrating molecular virology with cancer immunology. We conclude that the “diagnostic deficit” phenomenon was a reality during and immediately post-pandemic. However, a definitive answer to the questions related to the impact of the infection as an independent phenomenon would require advanced research information covering the biology of the viral infection and lung cancer oncogenesis: processes that are not currently implemented in routine clinical laboratory investigations.

## 1. Introduction

The COVID-19 pandemic triggered a sudden and profound disruption of oncology services worldwide, with multiple population-based registries documenting drastic declines in cancer diagnoses during 2020–2022 [1,2]. Among the conditions most visibly affected was lung cancer (LC), which remains the leading cause of cancer-related death worldwide, accounting for approximately 1.8 million deaths annually [2,3,4,5,6,7,8,9] (Table 1). In the first pandemic year, several independent national and regional cancer registries reported striking reductions in incident lung cancer diagnoses compared with 2019, with estimates ranging from a 10 to 20% decline in overall case numbers in the United States, United Kingdom, Italy, Poland, and Japan [5,6,7,8,9].

**Figure 1:**
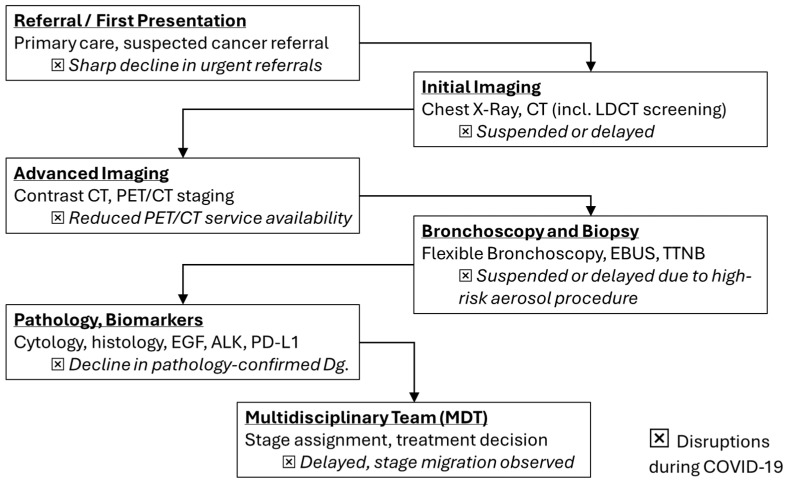
Lung cancer diagnostic flowchart with highlighted disruptions (⮽) during COVID-19 (adapted from concepts from [10,11,12,13,14,15,17,18,19,20,21]).

These incidence troughs coincided with health-system reorganization, curtailed elective imaging, restricted aerosol-generating diagnostics such as bronchoscopy, and widespread patient avoidance of healthcare settings. The phenomenon was a “diagnostic deficit” rather than a true fall in carcinogenesis [10,11,12,13,14,15,16]. In accordance with this interpretation, datasets from the United States, Europe, and Asia report stage migration toward advanced disease without commensurate reductions in mortality, implying delayed recognition of early-stage tumors that typically confer curative opportunities [15,17,18,19,20,21,22,23] (Figure 1).

Beyond service disruption, SARS-CoV-2 biology plausibly intersects with lung oncogenesis through host–virus pathways that regulate epithelial injury, inflammation, and immune surveillance [24,25,26,27,28,29,30]. Viral entry via ACE2 and priming by TMPRSS2 initiate a replication program, the proteins of which modulate autophagy, inflammasome activation, and antigen presentation. These mechanisms may be linked to tumor promotion and immune evasion in thoracic oncology [24,25,31,32,33,34,35,36,37,38,39,40,41]. Acute COVID-19 features an imbalanced host response marked by elevations of IL-1β, IL-6, IL-8, TNF-α and interferon-inducible chemokines (e.g., CXCL9/CXCL10) that govern myeloid recruitment, T-cell positioning, angiogenesis, and tissue remodeling. These axes have established roles in lung-cancer progression and checkpoint responsiveness [39,40,41,42,43,44,45,46,47,48,49,50,51,52]. Long COVID further reveals persistent innate activation and cytokine/chemokine dysregulation, including the IL-1β/IL-6/TNF-α triad and perturbations in CCL2, CCL5, and CXCL9. These changes also raise the possibility that chronic post-viral inflammation could condition pro-tumor microenvironments in susceptible hosts [53,54,55,56,57,58,59,60,61,62,63,64,65].

At the same time, current population-level analyses largely attribute pandemic-era incidence fluctuations to diagnostic backlogs and reporting dynamics rather than to virus-driven changes in tumor initiation, highlighting a critical need to disentangle service-delivery artifacts from any genuine biological effects [5,6,15,17,21,66].

This review presents and synthesizes data evidence across epidemiology as published by the Global Burden of Disease 2019 Cancer Collaboration, Global Cancer Statistics (GLOBOCAN), the International Agency for Research on Cancer (IARC), and the International Association of Cancer Registries (IACR) for virology, lung-cancer pathogenesis, immune-checkpoint biology, acute and post-acute immune dysregulation, and hypothesized oncogenic mechanisms of SARS-CoV-2 as published between 2019 and 2025 [5,7,17,20,45,46,47,48,67,68,69,70,71]. We aim to discuss whether observed declines in lung-cancer diagnoses predominantly reflect temporary diagnostic disruption or reveal deeper intersections between COVID-19 and pulmonary oncogenesis with implications for screening, diagnosis, and therapeutic strategy.

## 2. The Observed Effect of the COVID-19 Pandemic on Cancer Incidences

The onset of the COVID-19 pandemic in early 2020 was associated with an abrupt global decline in newly diagnosed cancer cases, a trend documented across multiple national cancer registries and hospital networks [5,6,7,8,9,13,14,15]. In the United States, analysis of the U.S. Cancer Statistics dataset covering nearly 97% of the population demonstrated a 13.6% reduction in age-adjusted lung cancer incidence between 2019 and 2020, falling from 47.9 to 41.4 cases per 100,000 [17]. Importantly, this reduction was accompanied by a relative increase in late-stage diagnoses, consistent with delayed recognition of early-stage disease rather than a true fall in occurrence [17,18].

Similar disruptions were reported across Europe. In the United Kingdom, urgent cancer referrals dropped by more than 60% in April 2020, coinciding with the suspension of routine imaging and drastic reductions in bronchoscopy volumes [18,19]. LC was particularly affected because its diagnostic pathway relies heavily on chest radiography, CT scans, and invasive procedures that were deprioritized due to their aerosol-generating potential [10,16,19]. In Italy, registry data documented a marked decline in LC diagnoses during 2020, with stage shifts toward more advanced disease [20]. Polish and Scandinavian registries reported comparable deficits, highlighting that the problem was not limited to a single healthcare system but represented a widespread phenomenon [21,72].

In Japan, nationwide surveys showed reductions in cancer surgeries, diagnostic imaging, and pathology-confirmed lung cancer cases, again concentrated in early stages [73]. In South Korea, where public health controls limited healthcare disruption, the decline in new cancer diagnoses was smaller but still measurable, suggesting that more effort is required to prevent diagnostic deficits [74].

Canadian and U.S. community oncology networks also described a collapse in pathology report volumes and delayed treatment initiation, with LC showing among the steepest deficits [15,75].

**Figure 2:**
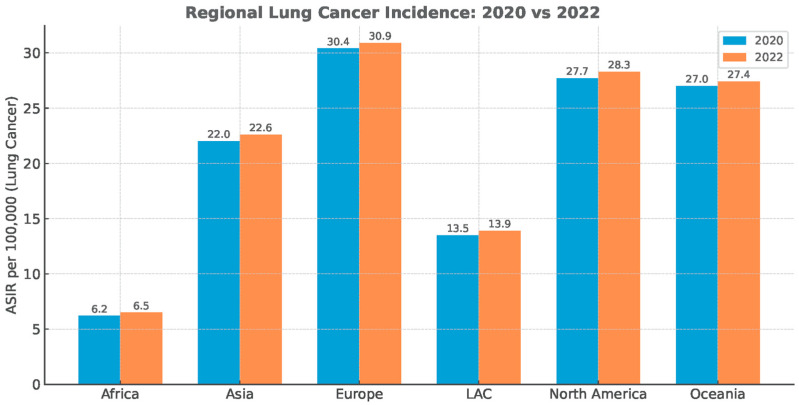
Regional lung cancer age-standardized incidence rate (ASIR). Data Source: Global Cancer Statistics (GLOBOCAN), simplified for visual representation.

The diagnostic deficit did not recover, despite the substantial reduction in anti-epidemiologic measures immediately post-pandemic (Figure 2).

The Romanian experience mirrored these international trends but was even more pronounced due to structural vulnerabilities in the healthcare system. During 2020, the addressability of patients to medical services fell drastically, driven both by fear of SARS-CoV-2 infection and by the reorganization of hospitals into COVID-only facilities. Reports from pulmonology departments indicated that bronchoscopy services were almost entirely suspended nationwide, as the procedure was considered high risk for aerosol generation and viral spread [16]. This meant that for much of the first pandemic year, patients with suspicious radiological findings had no access to diagnostic confirmation, creating a near-collapse in the LC diagnostic pipeline. Even in tertiary centers, bronchoscopy lists were reduced to emergency procedures, and the majority of suspected cases were deferred indefinitely [16,76]. As a result, Romanian oncologists observed substantial reductions in new LC case reporting during 2020–2021, consistent with the “diagnostic deficit” phenomenon described elsewhere.

**Figure 3:**
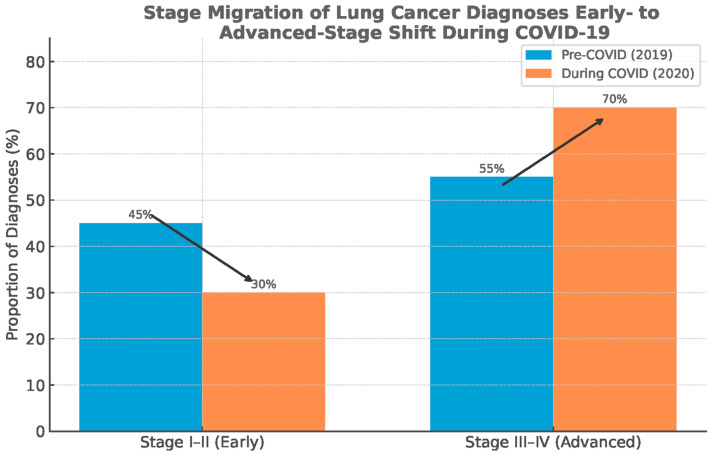
Stage migration: shift from early-stage to advanced-stage diagnosis of lung cancer. Data source: Global Cancer Statistics (GLOBOCAN), simplified for visual representation.

Globally, several consistent patterns emerge. First, the temporal nadir in cancer diagnoses closely matched the timing of lockdowns, healthcare reorganizations, and procedural suspensions [13,18,20]. Second, the deficit was disproportionately concentrated in early-stage and screen-detected cancers, while symptomatic late-stage cases were more likely to still present, producing a relative enrichment of advanced disease in 2020 datasets [15,17,21]. Third, despite partial recovery of case numbers that occurred in 2021, the rebound was incomplete, and there was no compensatory overshoot in diagnostic activities to account for the missing cohort of 2020 cases (Figure 3) [13,21,72].

Healthcare system analyses attribute these trends to several converging mechanisms: suspension of elective imaging and procedures, reallocation of oncology staff to COVID-19 wards, reduced operating room capacity for cancer surgery, and patient-level avoidance of healthcare settings due to fear of contagion [10,11,12,13,19].

The implications for survival outcomes are significant. Modeling studies suggest that even a three-month diagnostic delay can reduce LC survival by allowing stage progression. Furthermore, widespread year-long diagnostic deficits are expected to translate into measurable increases in cancer mortality in the coming decade [22,67]. Some analyses [14,17,23] estimate that the combined effects of diagnostic delays and treatment interruptions could result in thousands of excess overall cancer deaths across Europe and North America, with LC contributing disproportionately due to its rapid natural history.

Indeed, the convergence of data across continents, including the USA, Romania, Scandinavian countries, Italy, Japan, and the UK, provides compelling evidence that the pandemic precipitated a global diagnostic deficit, sharply reducing observed LC incidence in 2020 while paradoxically worsening prognosis [5,7,16,17,18,21].

## 3. SARS-CoV-2 Infection, Transmission, Replication–Transcription, Proteins, and Treatment

SARS-CoV-2 is an enveloped, positive-sense single-stranded RNA beta coronavirus whose entry into human cells is mediated primarily by the spike (S) glycoprotein engaging the angiotensin-converting enzyme 2 (ACE2) receptor [24,31,32]. Cryo-EM and crystallographic studies resolved the prefusion trimeric S architecture and the receptor-binding domain (RBD) complexed with ACE2, establishing the structural basis for high-affinity attachment and conformational transitions required for membrane fusion [31,32].

Following receptor engagement, S is primed by host proteases (notably TMPRSS2 and cathepsins) to trigger fusion at the plasma membrane or within endosomes, initiating cytosolic release of the ~30 kb RNA genome [24].

Once internalized, the viral replicase is translated from the genomic RNA to yield polyproteins pp1a/pp1ab, which are cleaved by the main protease (Mpro/3CLpro) and papain-like protease (PLpro) into nonstructural proteins (nsps) that assemble the replication-transcription complex (RTC) [25].

**Figure 4:**
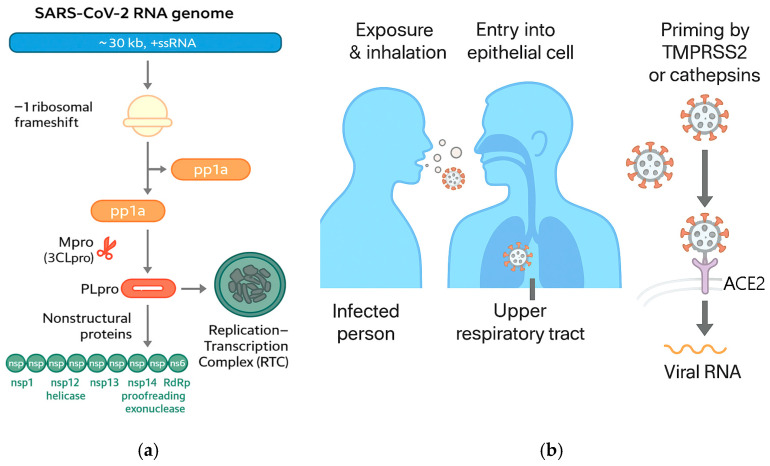
(**a**) SARS-CoV-2 RNA genome, replication and transcription; (**b**) COVID-19 transmission (adapted from concepts from [24,25,26,31,32,33,34,35,36,37,38,39,40,41,42]).

The RTC forms on virus-induced double-membrane vesicles where RNA synthesis proceeds, producing negative-sense intermediates and a nested set of sub-genomic mRNAs that encode structural (S, E, M, N) and accessory proteins [24,25]. Host–virus interactome mapping has identified numerous cellular pathways hijacked by the RTC and accessory proteins, including translation control, vesicular trafficking, and innate immune sensing hubs [26] (Figure 4).

Multiple viral proteins actively remodel intracellular trafficking and degradative pathways to favor replication while dampening antigen presentation [24,26]; the accessory protein ORF3a inhibits autophagosome–lysosome fusion by interacting with the HOPS tethering complex (e.g., VPS39), thereby blocking autophagic flux and potentially prolonging survival of infected cells [33,34]. Nsp6 provokes NLRP3 inflammasome activation and pyroptosis via ATP6AP1 targeting, illustrating a direct link between specific viral components and inflammatory cell death programs [35,36]. ORF8 down-regulates MHC-I on the host cell surface, a strategy that impairs antigen presentation to CD8^+^ T cells and facilitates immune evasion [37,38].

The characteristic host response to acute SARS-CoV-2 infection features blunted type I/III interferon signals juxtaposed with exuberant pro-inflammatory cytokine and chemokine production [39,40]. Studies identified IL-6, IL-8 (CXCL8), and TNF-α as independent predictors of in-hospital mortality, underscoring their central role in COVID-19 pathophysiology and triage algorithms [41].

Meta-analyses and multicenter datasets further associate severe disease with elevations in CCL2/MCP-1, CXCL9/MIG, and CXCL10/IP-10, chemokines linked to monocyte and CXCR3^+^ T-cell trafficking that correlate with respiratory failure [42,43,44].

These inflammatory signatures converge with the cellular reprogramming described above, in which viral proteins alter autophagy, inflammasome activation, and antigen presentation, together shaping a permissive yet tissue-damaging milieu in the lung [33,34,35,36,37,38,39,40,42,43,44]. Beyond canonical cytokines, chemokine perturbations have mechanistic and therapeutic implications in COVID-19 and bear relevance for thoracic oncology [42,43,44]. Elevations of CCL2/MCP-1 reflect monocyte recruitment programs that are also prominent in tumor-associated macrophage (TAM) biology, suggesting shared axes of immune-cell trafficking in viral pneumonia and lung cancer microenvironments [42,45,46,77]. In severe COVID-19, increased CXCL9 and CXCL10 track with IFN-γ-driven inflammation and predict adverse trajectories, paralleling their known roles in orchestrating CXCR3^+^ effector T-cell positioning in tumors and responses to immune checkpoint blockade [42,43,44,47,48].

These overlaps between antiviral and antitumor chemokine networks provide a conceptual bridge for later sections examining immune-checkpoint dysfunction in lung cancer and the long-term immune dysregulation after SARS-CoV-2 infection [42,43,44,47,48].

Transmission dynamics of SARS-CoV-2 are driven by high viral loads in the upper respiratory tract and efficient droplet/aerosol spread, with peak infectiousness near symptom onset and in the presymptomatic window [31].

Although successive variants altered intrinsic transmissibility and immune escape, the fundamental entry, replication, and many antagonism strategies remained conserved, maintaining therapeutic relevance of targets such as Mpro and the viral polymerase (RdRp/nsp12) [24,25]. Therapeutic development rapidly translated mechanistic insights into effective antivirals and immunomodulators [24,25,26].

The ACTT-1 randomized trial showed that remdesivir, an RdRp inhibitor, shortened time to recovery among hospitalized adults with lower respiratory tract involvement versus placebo [78]. A large platform trial (RECOVERY) demonstrated that low-dose dexamethasone reduced 28-day mortality among patients requiring oxygen or mechanical ventilation, establishing corticosteroids as a standard of care for hypoxemic COVID-19 [79]. In critically ill patients, interleukin-6 receptor antagonists (tocilizumab or sarilumab) improved outcomes when added to standard care, supporting targeted cytokine modulation in hyperinflammation [80].

For outpatients at high risk of progression, oral nirmatrelvir–ritonavir (a potent Mpro inhibitor co-formulated with a pharmacokinetic enhancer) significantly lowered the risk of hospitalization or death when initiated early [81].

Finally, virally induced tissue remodeling may intersect with cancer risk biology, though definitive causal links remain unproven [24,27,39].

Reviews and hypothesis publications have posited that persistent inflammation, fibrosis, and ACE2 pathway perturbations could, in theory, influence carcinogenic processes in the lung. However, longitudinal population-level evidence directly tying SARS-CoV-2 infection to increased lung cancer incidence is currently lacking [27,28,29,30].

Given this uncertainty, it is essential to distinguish between the observed pandemic-era fall in lung cancer incidence due to diagnostic disruption and the speculative notion of virus-driven oncogenesis. This subject will be discussed again after detailing LC pathogenesis and immune dysregulation.

## 4. Pathogenesis of Lung Cancer

### 4.1. Driving Oncogenic Mutations Define the Histological Characteristics of the Tumor

LC encompasses histologically and molecularly distinct entities: predominantly non-small-cell lung cancer (NSCLC adenocarcinoma, squamous, and large-cell types) and small-cell lung cancer (SCLC), which arise through accumulated genomic alterations, epigenetic reprogramming, and selection within inflamed or injured lung microenvironments [82].

Comprehensive atlases delineate the recurrent driver-mutation landscape in NSCLC, with LUAD (Lung Adenocarcinoma) enriched for KRAS, EGFR, BRAF, MET exon 14 skipping, and kinase fusions (ALK, ROS1, RET), and LUSC (Lung Squamous Cell Carcinoma) dominated by TP53 loss, CDKN2A inactivation, SOX2/FGFR1 amplifications, and oxidative-stress pathway lesions (KEAP1–NFE2L2) [83,84].

Tobacco smoke produces characteristic cytosine-to-adenine base substitutions and a high tumor mutational burden, whereas never-smokers more often harbor oncogene-related LUAD with distinct etiologies, highlighting exposure–genotype interactions [85].

Environmental and occupational cofactors, including indoor radon, silica dust, asbestos, and ambient fine particulates (PM2.5), make independent contributions to LC risk beyond active smoking, with dose–response relationships documented in large cohorts and meta-analyses [86,87,88,89].

### 4.2. Co-Mutations Shape Immune Contexture and Therapy Response [82,83,84]

In KRAS-mutant LUAD, the co-loss of STK11/LKB1 or KEAP1 defines “immune-cold”, myeloid-skewed tumors with low PD-L1 and attenuated responses to PD-(L)1 blockade, a phenotype reproduced across retrospective cohorts and translational datasets. By contrast, KRAS tumors lacking these co-alterations more often exhibit inflammatory T-cell infiltration (“immune hot”) and can respond favorably to checkpoint inhibitors, underscoring the interaction between tumor-intrinsic circuitry and antitumor immunity [34,90].

### 4.3. Cytokine Signaling Is Integral to Lung Tumor Progression [82]

The IL-6–JAK–STAT3 pathway promotes survival, stemness, epithelial-to-mesenchymal transition (EMT), metabolic reshaping, and immunosuppression via induction of PD-L1 and myeloid recruitment programs, placing IL-6 at the nexus of cancer cell fitness and microenvironmental control [49]. IL-8/CXCL8, produced by tumor cells, fibroblasts, and myeloid cells, drives neutrophil chemotaxis, angiogenesis, and EMT, correlating with aggressive disease and resistance to targeted and immune therapies [50].

IL-1β licensing of myeloid cells fosters angiogenesis and suppressive macrophage/MDSC accumulation. Exploratory analysis of inhibition of IL1β by Canakinumab (CANTOS trial) has been shown to significantly reduce lung cancer incidence and mortality. The findings strengthen the mechanistic link between chronic IL-1 signaling and lung tumorigenesis, as suggested by delayed progression of diverse molecular subtypes of lung cancer subsequent to reduction in tumor-promoting inflammation [51,52].

TNF-α is pleiotropic in cancer but chronically activates NF-κB and AP-1 programs that support proliferation, invasion, and EMT, with context-dependent crosstalk that can tip inflammation toward tumor promotion [91].

### 4.4. Chemokine Circuits Orchestrate Cellular Traffic and Functional States in the Tumor Microenvironment

CCL2/CCR2 recruits inflammatory monocytes that differentiate into tumor-associated macrophages (TAMs), often assuming immunosuppressive, pro-angiogenic phenotypes that blunt cytotoxic T-cell control [45,46]. CCL5/CCR5 signaling is context-dependent, supporting CD4^+^/CD8^+^ recruitment under effective priming yet also attracting CCR5^+^ macrophages and Tregs that promote immune escape, helping explain heterogeneous correlations with outcomes across datasets [46,47,77].

Conversely, interferon-inducible CXCL9/CXCL10 guide CXCR3^+^ effector T cells into tumors and associate with benefit from PD-1/PD-L1 blockade in preclinical models and human translational studies, linking type-I/II interferon tone to checkpoint responsiveness [47,48].

### 4.5. Tissue Context Is Important for Tumorigenesis and Disease Progression

Patients with idiopathic pulmonary fibrosis (IPF) and other fibrotic interstitial lung diseases have a markedly increased incidence of lung cancer, illustrating how repetitive injury, aberrant repair, and profibrotic cytokines create permissive niches for oncogenesis that may be recapitulated after severe viral pneumonitis [92].

Because staging as part of diagnosis is the dominant determinant of treatment and subsequent survival in lung cancer, even brief perturbations to imaging and tissue acquisition (such as pandemic-era interruptions) translate into stage advancement and worse outcomes, independent of any change in the underlying rate of carcinogenesis [67,82].

In sum, lung cancer pathogenesis reflects the integration of carcinogen-driven genomics with inflammation-shaped microenvironment, wherein IL-1β, IL-6, IL-8, TNF-α, and chemokines CCL2, CCL5, CXCL9 tune angiogenesis, myeloid skewing, T-cell response positioning, EMT, and checkpoint responsiveness, providing multiple mechanistic bridges to the cytokine–chemokine perturbations observed in acute and post-acute COVID-19 [45,46,47,48,49,50,52,82,83,84,85,90,91,92]. Table 2 presents possible cytokine/chemokine functions in LC pathogenesis.

## 5. Dysfunction of the Immune System in Lung Cancer: The Role of Immune Checkpoints

Physiological immune checkpoints such as CTLA-4 and PD-1/PD-L1 enforce immune tolerance and limit tissue damage during inflammation and tissue repair. Tumors exploit these pathways to attenuate T-cell priming and effector function within the tumor microenvironment (TME) [91].

In NSCLC, treatments with monoclonal antibodies that block PD-1 or PD-L1 durably improve objective response rate (ORR), progression-free survival (PFS), and overall survival (OS) compared with standard platinum chemotherapy in biomarker-selected individuals [93,94,95,96,97]. First-line pembrolizumab significantly prolonged OS versus chemotherapy in metastatic NSCLC with PD-L1 tumor proportion score (TPS) ≥ 50%, with a 5-year OS rate of 31.9% versus 16.3% and sustained tolerability [93].

Dual checkpoint blockade with nivolumab plus ipilimumab improved OS and PFS compared with chemotherapy across PD-L1 strata in the CheckMate 227 trial, establishing CTLA-4 co-inhibition as a viable option for selected patients [94].

Atezolizumab monotherapy (IMpower110) extended OS in treatment-naïve metastatic NSCLC with high PD-L1 expression, confirming PD-L1 as an enrichment biomarker for anti-PD-(L)1 benefit [95].

Cemiplimab likewise improved survival in EMPOWER-Lung 1 for PD-L1 ≥ 50% NSCLC, with durable benefit in longer follow-up analyses [96,97] (Table 3).

Checkpoint expression is shaped by tumor-intrinsic oncogenic programs and inflammatory tone that govern antigenicity, interferon signaling, and myeloid composition [82,90]. KRAS-mutant LUAD co-altered by STK11/LKB1 or KEAP1 mutations exhibits a myeloid-dominant, “cold tumor” state with low PD-L1 and inferior responses to PD-(L)1 therapy [90].

Pro-inflammatory cytokines relevant to pulmonary oncology intersect with checkpoint pathways: IL-6 trans-activates JAK/STAT3 in lung cancer and can upregulate PD-L1, while EGFR signaling on tumor cells converges on the IL-6–STAT3 axis to enhance PD-L1 expression [98,99]. IL-8/CXCL8 promotes neutrophil influx and EMT and predicts checkpoint inhibitor treatment resistance. Experimental IL-8 blockade restores anti-PD-1 activity in preclinical models [100,101,102].

Type-II interferon (IFN-γ) inflammatory signaling induces PD-L1 expression on cancer cells, conducive for tumor immune evasion, and at the same time upregulates chemokines such as CXCL9/CXCL10 that recruit CXCR3^+^ effector T cells as a cytotoxic response to cancer [47,48].

Axes such as CCL2/CCR2 and CCL5/CCR5 regulate tumor-associated monocyte/macrophage (TAM) and regulatory T-cell (Treg) trafficking, often fostering immunosuppressive niches that hinder cytotoxic lymphocyte (CTL) function, whereas CXCL9/CXCL10 support CTL recruitment and are associated with benefit from PD-1/PD-L1 blockade [45,46,47,48] (Table 4).

Extensive checkpoint inhibition may cause immune-related adverse events (irAEs) that reflect systemic disinhibition of immune responses. Among pulmonary toxicities, checkpoint inhibitor pneumonitis (CIP) is uncommon but potentially life-threatening, with higher incidence in lung cancer patients and combination regimens [103,104,105,106]. Not surprisingly, radiological ground-glass opacities and organizing-pneumonia patterns predominate in CIP but overlap with viral pneumonias such as COVID-19, having exacerbated immune response as a common background and necessitating clinical and microbiologic adjudication and, when needed, bronchoscopy [107,108,109].

During the pandemic, imaging similarities between CIP and COVID-19 pneumonia complicated triage and management, prompting multidisciplinary algorithms incorporating exposure history, PCR testing, timing relative to ICI doses, and, when feasible, bronchoscopy with protected specimen collection [107,108,109].

**Figure 5:**
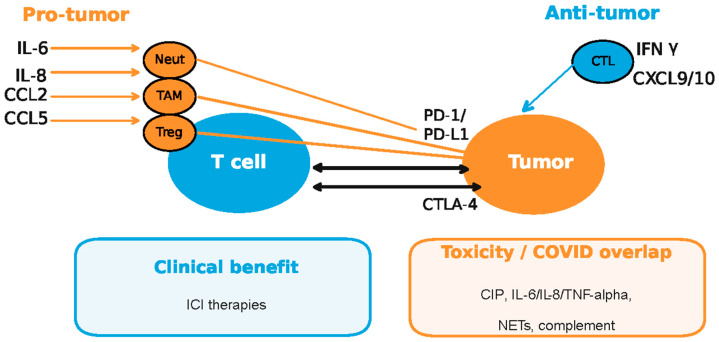
Pro-tumor and antitumor responses in the context of COVID-19 dysregulated cytokine/chemokine circuits and checkpoint inhibitors (ICI—immune checkpoint inhibitors; CIP—checkpoint inhibitor pneumonitis; CTL—cytotoxic T lymphocytes; TAM—tumor-associated macrophages; Neut—neutrophils; NETs—neutrophil extracellular traps). Enhanced cytokine secretion by myeloid descendants (neutrophils, macrophages and dendritic cells) and regulatory T cells (Tregs) during the viral infection is known to cause the upregulation of checkpoints on tumor cells conductive to immune escape, while CTL cell-mediated anti-viral response by IFNg secretion exposes the tumor to increased tumor-associated antigen (TAA) recognition and CTL attack (adapted from concepts from [45,46,47,48,90,93,94,95,96,97,98,99,100,101,102]).

Together, these data place immune checkpoints at the heart of lung cancer immune dysfunction—where cytokine/chemokine circuits (e.g., IL-1β, IL-6, IL-8, TNF-α; CCL2, CCL5, CXCL9) modulate T-cell responses, myeloid skewing, and PD1/PD-L1 regulation—and illustrate how pandemic-related diagnostic barriers intersected with an already complex immunobiology [45,46,47,48,90,93,94,95,96,97,98,99,100,101,102] (Figure 5).

Theoretically, the overlapping immunologic enhancement of COVID-19 infection and ICI therapy could result in an increase in immune-related adverse events (irAEs). A single-center study suggested that COVID-19 may pose a risk of severe irAEs in cancer patients receiving ICIs. Close monitoring and possibly delaying ICI administration could be considered when cancer patients are infected with COVID-19. The study included, besides NSCLC (the majority of the cases), other tumor types, including renal cell carcinoma, melanoma, hepatocellular cancer, endometrial cancer, and uterine cancer. The incidence of irAE was higher in COVID-19 positive patients (30.4% vs. 19.9%), but it was not statistically significant (*p* = 0.13). On the other hand, a significantly higher incidence of severe irAEs was noted in the COVID-19 group (10.9% vs. 3.2%, *p* = 0.02) [110]. In a different study that included 159 patients treated with ICIs, in which 52 patients (32.7%) were infected with COVID-19, the authors found the incidence of AEs was not increased in the COVID-19 positive group treated with either ICIs, chemotherapy, or targeted therapy (*p* = 0.22), and there was no apparent increased risk of irAEs in the COVID-19 infected patients [111].

## 6. Immune Dysregulation in Acute SARS-CoV-2 Infection

Acute COVID-19 is characterized by an imbalanced host response: attenuated type-I/III interferon signaling alongside exuberant pro-inflammatory cytokine and chemokine production that correlates with respiratory failure [39,40,41,43,44]. Key mediators include IL-6, IL-8 (CXCL8), TNF-α, and interferon-inducible chemokines (CXCL9/MIG, CXCL10/IP-10) that drive monocyte recruitment, neutrophil activation, and CXCR3^+^ T-cell trafficking into inflamed lungs [41,43,44]. High IL-6, IL-8, TNF-α, and CXCL10 identify high-risk trajectories and inform triage and monitoring strategies [41,43,44]. Clinically, these axes justify targeted therapies: low-dose dexamethasone to dampen host-mediated lung injury; IL-6 receptor antagonists in critically ill patients; and targeted antivirals (remdesivir, nirmatrelvir–ritonavir) to curb viral replication and reduce inflammatory burden [78,79,80,81].

Elevated CCL2/MCP-1 mirrors monocyte-mobilizing programs also implicated in tumor-associated macrophage biology, highlighting mechanistic overlaps between acute viral pneumonitis and cancer microenvironments [42,45,46].

Pathology and translational studies reveal diffuse alveolar damage coupled to pulmonary endotheliosis, platelet–fibrin microthrombi, and intussusceptive angiogenesis, distinguishing severe COVID-19 from typical ARDS and implicating immunothrombosis in hypoxemia [112,113,114].

Neutrophil extracellular traps (NETs) are markedly increased in severe disease and associate with thrombosis and organ damage, positioning NET-osis as a therapeutic target [115,116,117]. Complement activation contributes to microvascular injury, with deposition of C4d and C5b-9 in lung and cutaneous vessels in severe COVID-19 and broader evidence of complement–coagulation crosstalk [118,119,120].

Viral proteins interface with innate immunity to potentiate this inflammatory milieu [24,25,26,33,34,35,36,39]. Nsp6 triggers NLRP3-dependent pyroptosis via ATP6AP1 targeting, while ORF3a impairs autophagosome–lysosome fusion, thereby sustaining danger signaling and antigen persistence [33,35]. ORF8 down-regulates MHC-I, hindering antigen presentation to CD8^+^ T cells and blunting cytotoxic clearance of infected cells [37,38].

**Figure 6:**
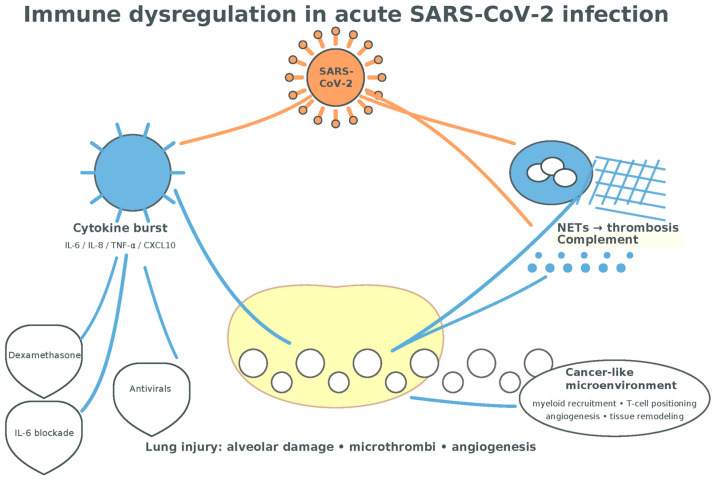
Immune dysregulation in acute SARS-CoV2 infection. The cytokine burst following T-cell-mediated inflammation associated with myeloid cells-mediated NETosis is presumptively conductive to a tumorigenic environment (alveolar damage, neo-angiogenesis, tissue remodeling, and fibrosis) (adapted from concepts from [35,36,37,38,39,40,41,42,43,44,45,46,78,79,80,115,116,117,118,119,120]).

Collectively, acute COVID-19 generates a cytokine- and chemokine-rich environment (IL-1β, IL-6, IL-8, IL-17, TNF-α, CCL2, CCL5, CXCL9) that overlaps with lung-cancer-relevant circuits governing myeloid recruitment, T-cell positioning, angiogenesis, and tissue remodeling, setting the stage for the longer-term immune perturbations discussed next [42,43,44,45,46,47,48,49,50,51,52,115,116,117,118,119,120] (Figure 6).

## 7. Immune Dysregulation in Long COVID

Long COVID (post-acute sequelae of SARS-CoV-2, PASC) is characterized by persistent immune activation and a lack of coordinated adaptive responses months after infection [53]. Deep immunophenotyping demonstrates skewed T-cell subset distributions, an increase in CD4^+^ T cells with tissue-homing phenotypes, and exhausted SARS-CoV-2-specific CD8^+^ T cells in individuals with long COVID compared with convalescent controls [53].

These perturbations co-exist with higher anti-SARS-CoV-2 antibody levels and a miscoordination between virus-specific T- and B-cell responses, implying continued inflammatory stimuli or antigen persistence [53]. Furthermore, independent cohorts confirm that immunological dysfunction, including elevated inflammatory mediators and altered innate and adaptive immune cell populations, can persist for at least eight months following mild-to-moderate infection [54].

Multi-omic analyses during acute infection identify risk signatures for PASC, including SARS-CoV-2 nucleocapsid (N) protein antigenemia, RNAemia, specific autoantibodies, and herpesvirus (e.g., EBV) reactivation, which predict downstream immune dysregulation and symptom persistence [55].

Across studies, a reproducible cytokine signature featuring interleukin-1β (IL-1β), interleukin-6 (IL-6), and tumor necrosis factor-α (TNF-α), the so-called “cytokine triad”, is associated with long COVID and correlates with ongoing symptom burden and distinguishes affected individuals from matched convalescent controls [56]. Subsequent longitudinal work supported persistent elevation of this triad months after infection and associated it with fatigue, exertional intolerance, and neurocognitive symptoms [57] along with immune cell phenotypes indicative of low-grade chronic inflammation [58,59,60].

Among well-established immune responses, Th17-mediated immunity appears variably engaged [121]. Some studies report that increased IL-17 family cytokines or Th17-skewing signatures in long COVID, especially in patients with neurocognitive symptoms, fatigue, or dysautonomia [121,122]. Importantly, reports of increased IL-17 (or IL-17F) are stronger in acute/severe COVID-19 and in analyses linking endothelial activation to persistent symptoms; nonetheless, causality for chronic pulmonary symptoms remains under study [123,124].

Chemokine axes that coordinate leukocyte trafficking also remain unsettled in long COVID [60]. Increased monocyte chemoattractant protein-1/CCL2 (MCP-1) has been observed in PASC cohorts, with some data suggesting later rises beyond one year in persistent cases [61]. The CCL2–CCR2 pathway is a recognized amplifier of COVID-19 lung inflammation during acute disease, and elevated CCL2 has been documented in respiratory specimens and blood of severe cases [62].

Reports in PASC also describe dysregulation of CCL5/RANTES and CXCR3 ligands (e.g., CXCL9/MIG), consistent with sustained trafficking signals for activated T cells and monocytes. Such patterns have been linked to symptom clusters and endothelial activation in post-COVID patients [60,63,64,65]. Machine-learning classifiers trained on cytokine/chemokine hubs (including CCL5 and IL-6) were shown to distinguish PASC from other chronic inflammatory conditions, underscoring the biologic specificity of these immune signatures [125].

## 8. Possible Oncogenic Mechanisms of SARS-CoV-2, Hypotheses and Evidence Gaps

No definitive epidemiologic evidence demonstrates that SARS-CoV-2 infection increases population-level LC incidence independent of diagnostic disruption, but multiple mechanistic hypotheses deserve careful evaluation [6,17,21,66]. On the other hand, SARS-CoV-2 proteins and host responses could intersect with the “hallmarks of cancer”, including sustained proliferative signaling, resistance to cell death, angiogenesis, immune evasion, and chronic inflammation [66,126,127].

**Figure 7:**
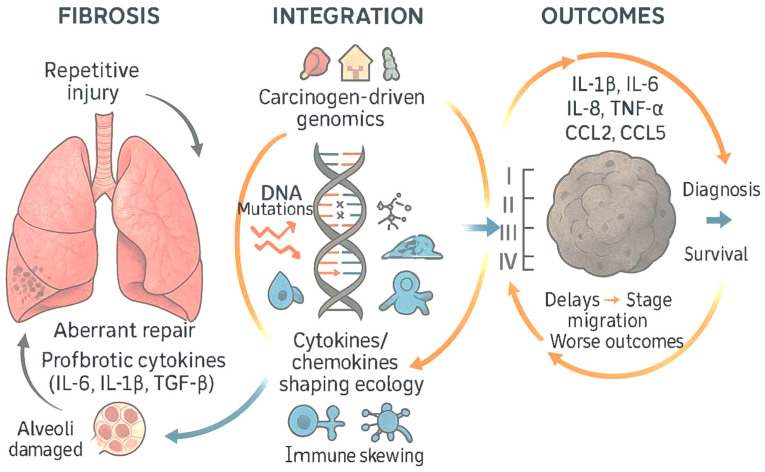
Epithelial–endothelial injury and aberrant fibrotic repair sustained by the cytokine–chemokine environment are both conducive to the immune escape of cells with cancerous mutations and accelerated progression of the tumoral disease with implications on the stage at diagnosis and survival expectation (adapted from concepts from [53,54,55,56,57,58,59,60,61,62,66,125,126,127,130]).

At the tissue level, pulmonary imaging and physiology reveal persistent small-airways disease and gas-transfer impairment in symptomatic convalescents [128,129]. Quantitative expiratory CT analyses show air trapping consistent with distal airway dysfunction, often independent of initial acute severity [128]. Hyperpolarized xenon-129 MRI demonstrates persistent abnormalities in alveolar–capillary gas exchange among non-hospitalized individuals with dyspnea months after infection [129]. These physiologic alterations coexist with systemic inflammatory signatures, suggesting that ongoing epithelial–endothelial injury and aberrant repair may be sustained by the cytokine–chemokine environment described above [53,56,128,129] (Figure 7).

A leading, indirect pathway is inflammation-driven tumor promotion, particularly through IL-6/JAK–STAT3, IL-1β/NLRP3, TNF-α/NF-κB, and ELR^+^ CXC chemokines (e.g., IL-8/CXCL8), which are all engaged during COVID-19 and have established roles in lung tumor biology [68,69,70,71,98,99,101,105,112,130,131,132,133,134,135,136,137,138,139,140,141,142] (Table 5).

Synthesis of these data supports a model in long-COVID patients in which chronic innate activation (IL-1β, IL-6, TNF-α) and immune-cell trafficking (CCL2, CCL5, CXCL9) maintain a low-grade inflammatory state, superimposed on T-cell mis-coordination and exhaustion [53,56,59,60,61,65], while these patients exhibit long-term radiographic and functional alterations, with heterogeneous recovery trajectories over 6–12 months and beyond [154].

Persistent activation of these inflammatory pathways could plausibly modify lung cancer risk in susceptible hosts, even if direct causality has not been established [68,69,70,71,98,99,101,105,112].

SARS-CoV-2 may also modulate epithelial barrier repair and cell-intrinsic stress pathways relevant to oncogenesis [66,126]. Autophagy blockade by the viral accessory protein ORF3a impairs autophagosome–lysosome fusion, potentially increasing oxidative stress and cytoplasmic DNA accumulation that feed pro-tumor inflammatory circuits [33,155,156]. SARS-CoV-2 ORF8 downregulates MHC class I, and N-linked glycan interactions contribute to antigen-presentation interference, which in theory could transiently reduce immune surveillance against incipient transformed clones [37].

ACE2 downregulation during infection perturbs the renin–angiotensin system (RAS), tipping the balance toward angiotensin II/AT1R signaling that promotes fibrosis, oxidative stress, and pro-inflammatory cytokine production in the lung microenvironment [28,29].

Chronic fibrotic remodeling of lung parenchyma (i.e., idiopathic pulmonary fibrosis) is a recognized risk factor for lung cancer, suggesting that post-viral fibrosis, if durable, could indirectly raise risk in a subset of patients [92,131,157]. Radiology and functional imaging studies after COVID-19 document persistent small-airways disease and gas-transfer abnormalities consistent with ongoing epithelial–endothelial injury that might, over time, create a pro-carcinogenic niche in susceptible individuals [128,129,154].

A separate, contentious hypothesis is viral genome integration [132]. One in vitro study proposed LINE-1–mediated reverse transcription and rare integration of SARS-CoV-2 sequences into host DNA, which could, in principle, create insertional mutagenesis or aberrant transcripts [132]. However, multiple independent investigations using long-read and whole-genome sequencing found no convincing evidence of SARS-CoV-2 integration in patient tissues, arguing that such events, if they occur at all in vivo, are extremely rare and unlikely to influence oncogenesis or clinical testing [133,134,135,136]. Thus, current data support inflammation-facilitated and repair-mediated mechanisms rather than direct viral insertion as plausible drivers of any long-term cancer-modifying effects [66,126,127,139,140,141,142].

## 9. Therapeutic Implications

Host–tumor interface pathways perturbed in COVID-19 overlap with therapeutic axes in lung cancer [68,98,105,109,130]. IL-6/JAK–STAT3 inhibitors, IL-1 blockers, and strategies targeting CCR2/CCR5 are under investigation in oncology and may, with careful selection, have dual relevance in patients with prior severe COVID-19 and inflammation-dominated tumor microenvironments [68,139,147,148,149,150,158].

Conversely, immune checkpoint pathways central to lung cancer (PD-1/PD-L1, CTLA-4) show altered ligand/receptor expression during and after SARS-CoV-2 infection, raising conceptual questions about timing and dosing of immunotherapy in patients with recent COVID-19 or persistent immune activation [112,113].

An interesting but speculative hypothesis suggests that the nonspecific activation of the immune system after viral infections could produce an antitumor effect through several mechanisms, including the bystander effect and molecular mimicry. Historically, in anecdotal cases, severe viral infections with high fever have been associated with spontaneous cancer remission, a phenomenon that has informed the development of modern immunotherapies using oncolytic viruses.

The hypothetical mechanism can be attributed to a robust tumor bystander T-cell response generated against a viral infection. The response is initially non-specific, receptor independent and cytokine-mediated (i.e., IFN gamma) that triggers tumor antigen release, and/or converts a “cold” tumor into a “hot” tumor type with the newly expanded CTL infiltration.

The cell-mediated antiviral response could become cross-reactive, meaning the T cells also recognize and attack tumor cells that express a similar-looking antigen or fragments. The neoantigenic recognition and activation in such events could theoretically result in long-term protection against cancer by creating an immunological memory that recognizes tumor-associated antigens. At the same time, cross-reactive antigenic viral fragments could be a source of post-viral autoimmune responses.

**Figure 8:**
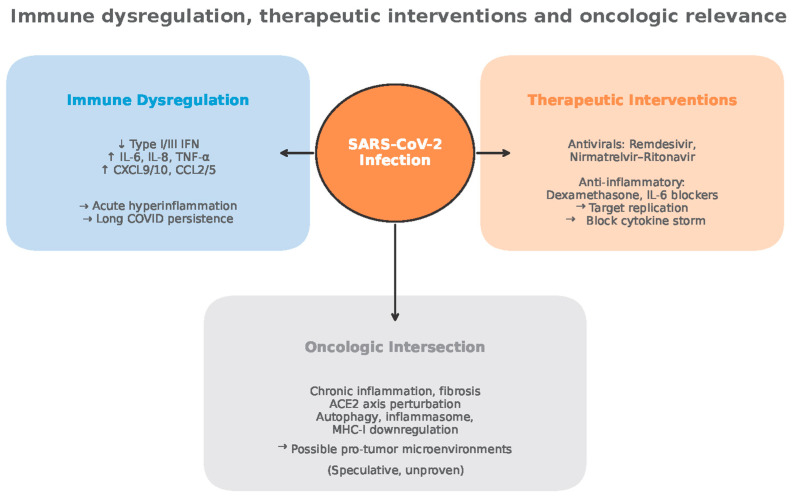
Immune dysregulation, therapeutic interventions and oncologic relevance (adapted from concepts from [28,29,30,53,54,55,56,59,60,61,65,82,98,105,112,113,121,122,123,124,125,128,132,133,134,135,136,154,159]).

Despite the possibility of antitumor effects, the therapeutic benefits of viral infection are not guaranteed and can be unpredictable [159] (Figure 8).

## 10. Discussion

Across registries and health-system cohorts, the temporal signature of LC diagnoses—a steep minimum in 2020 aligned with lockdowns, procedure suspensions, and patient avoidance—together with stage migration toward advanced disease, indicates that the observed incidence dip is chiefly a diagnostic artifact, not a true biologic suppression of carcinogenesis [4,6,8,160]. This pattern holds across the United States, United Kingdom, Italy, Poland, Scandinavia, Japan, Canada, and is mirrored in Romania, where professional guidance restricted bronchoscopy and addressability plummeted, underscoring common mechanisms with local nuances [4,8,10,11,12,17,18,19,72,160]. Health-services analyses implicate curtailed imaging, reallocated staff, reduced endoscopy/bronchoscopy throughput, and fear-driven care deferral, with incomplete rebounds in 2021 and no compensatory overshoot, implying that some “missing” early-stage cancers were never recaptured in registry counts [2,5,7,10,72].

However, SARS-CoV-2 biology plausibly intersects with lung cancer pathways via ACE2/RAS, autophagy/lysosomal stress, inflammasomes, and immune checkpoint circuits, with IL-1β, IL-6, IL-8, IL-17, TNF-α, CXCL9, CCL2, and CCL5 as shared effectors shaping both acute COVID-19 and lung tumor microenvironments [6,7,8,18,33,67,73]. In acute infection, these mediators track with severity and outcomes and provide targets for effective therapies (e.g., dexamethasone, IL-6R blockade) that reduce hyperinflammation, while oral antivirals lower progression risk and viral burden [35,36,37,38,39,46,47].

In Long COVID, persistent cytokine/chemokine dysregulation, T-cell exhaustion, monocyte activation, and fibrotic remodeling present credible pathways by which host immunity and tissue context could be tilted toward protumor states in vulnerable lungs, though rigorous epidemiology remains pending [6,7,8,9,17,20,73].

Medical practice implications follow on two fronts:

First, health-system recovery: prioritize catch-up low-dose radiological screening for eligible populations; restore and protect bronchoscopy capacity with optimal personal protection equipment and airflow; streamline rapid diagnostic pathways; and deploy equity-focused outreach to communities with the largest diagnostic shortfalls [10,13,17,18,19].

Second, updated research agenda: link infection history and vaccination status to cancer registries; embed biospecimen collection (airways and serum) in post-COVID cohorts; profile ACE2/RAS, autophagy/lysosome, NLRP3, and checkpoint axes; and validate cytokine/chemokine signatures (IL-6, IL-1β, IL-8, IL-17, TNF-α, CXCL9, CCL2, CCL5) as risk stratifiers or therapeutic adjuncts in pulmonary oncology [19,24,31,33,67].

These steps will differentiate diagnostic backlog from true biologic effects of respiratory infections, detect and quantify associated long-term risks, and ensure resilient cancer control during epidemic outbreaks.

## 11. Conclusions

The pandemic and post-pandemic decline in observed lung cancer diagnoses in 2020 is best explained by the indisputable health-system disruption, as substantiated by the advanced-stage diagnostic shift and incomplete rebounds to the pre-pandemic status quo, signaling a substantial diagnostic debt that threatens outcomes in the registered incidence and mortality of lung cancer [4,5,6,160].

Mechanistic plausibility exists for SARS-CoV-2 to modulate tumor–host ecosystems through fibrosis, persistent cytokine/chemokine dysregulation, and autophagy/antigen-presentation stress as a substrate for accelerated oncogenesis [19,24,31,33,67], not a reduction in oncogenesis.

The non-specific activation of bystander cellular immunity components and suppression of inhibitory immune circuits by SARS-CoV-2 (as well as other respiratory viral infections) that may resolve very early tumorigenic stages is a speculative hypothesis and requires substantial clinical and basic research data to be sustained.

The shared inflammatory circuits of viral infections and cancer warrant additional exploration. Multi-omics analyses and machine learning algorithms using accumulated data from multiple research institutions may elucidate whether, and how, COVID-19 shapes pulmonary oncogenesis over time.

From a public-health perspective, the anticipation of potential diagnostic deficits in lung cancer should be carefully considered for the potential downstream effects when considering the duration and structure of the quarantine protocols. After extensive quarantines for viral respiratory infections, such as those imposed during the COVID-19 pandemic, public health systems should support specialty clinicians to accelerate catch-up screening, safeguard diagnostic throughput, and maintain vigilant monitoring for inflammatory lung sequelae.

## Figures and Tables

**Table 1:** Global lung cancer (incl. trachea and bronchus) incidence and mortality (ASMR—age-standardized mortality rate; ASIR—age-standardized incidence rate).

Year	Deaths (Thousands)	ASMR per 100,000	Incidence (Thousands)	ASIR per 100,000	Data Source
2019	2040	25.2	2260	27.7	Global Burden of Disease 2019 Cancer Collaboration
2022	1818	16.8	2481	23.6	International Agency for Research on Cancer (IARC) and the International Association of Cancer Registries (IACR), CI5—Cancer Incidence in Five Continents

**Table 2:** Cytokine/chemokine functions in LC pathogenesis.

Molecule	Main Functions
IL-1β	Myeloid licensing, angiogenesis, EMT, immune suppression
IL-6	JAK–STAT3 signaling → survival, EMT, metabolic rewiring, PD-L1 induction, immunosuppression
IL-8 (CXCL8)	Neutrophil chemotaxis, angiogenesis, EMT, resistance to therapy
TNF-α	NF-κB/AP-1 activation, proliferation, invasion, EMT, tumor-promoting inflammation
CCL2 (MCP-1)	Monocyte recruitment → TAMs, immunosuppression, pro-angiogenic
CCL5	Dual role: T-cell recruitment vs. Treg/TAM immune escape
CXCL9 (±CXCL10)	IFN-inducible, recruits effector T cells, linked to PD-1/PD-L1 response

**Table 3:** Pivotal trials of PD-(L)1 and CTLA-4 blockade in metastatic NSCLC.

Study (Ref.)	Regimen	Setting/Population	PD-L1 Selection	Key Outcome (OS/Efficacy)	Notes
KEYNOTE-024 ([76])	Pembrolizumab vs. platinum chemo	First-line, metastatic NSCLC	TPS ≥ 50%	5-yr OS 31.9% vs. 16.3% (chemo); durable benefit	Established PD-L1 ≥ 50% as monotherapy indication
CheckMate 227 ([77])	Nivolumab + Ipilimumab vs. chemo	First-line, metastatic NSCLC	All PD-L1 strata	Improved OS vs. chemo across PD-L1 strata	Dual PD-1 + CTLA-4 blockade as option
IMpower110 ([78])	Atezolizumab vs. chemo	First-line, metastatic NSCLC	High PD-L1	Extended OS vs. chemo in PD-L1-high	Validated PD-L1 as enrichment biomarker
EMPOWER-Lung 1 ([80])	Cemiplimab vs. chemo	First-line, metastatic NSCLC	TPS ≥ 50%	Improved OS with durable benefit on longer follow-up	Supports anti-PD-1 monotherapy in PD-L1 ≥ 50%

**Table 4:** Tumor-intrinsic drivers and cytokine/chemokine circuits that shape ICI response.

Factor	Mechanistic Impact
KRAS + STK11/LKB1 or KEAP1 co-alterations	Immune-cold, myeloid-dominant TME; low PD-L1; inferior PD-(L)1 responses
IFN-γ → PD-L1 + CXCL9/10	T-cell-inflamed gene programs; improved checkpoint responsiveness
IL-6 → JAK/STAT3 (±EGFR input)	PD-L1 upregulation; immunosuppression; resistance circuits
IL-8 (CXCL8)	Neutrophil influx; EMT; resistance; high circulating IL-8 predicts poor ICI outcomes
CCL2/CCR2; CCL5/CCR5	Monocyte/macrophage & Treg trafficking → suppressive TAM niches
CXCL9/10	Recruitment of CXCR3^+^ effector T cells; associated with benefits for PD-1/PD-L1 blockade

**Table 5:** Cytokines highly secreted in COVID-19 with a potential role in lung tumorigenesis.

Inflammatory Cytokines	Potential Involvement in Tumorigenicity
IL-6 activates	Activates JAK–STAT3 signaling in epithelial and immune cells to support proliferation, survival, angiogenesis, and immunosuppression in lung cancer models [68,130,143]. Preclinical studies show that IL-6 blockade reduces lung tumor promotion and STAT3 activation in KRAS-mutant models, linking airway inflammation to tumor growth [143].
IL-1β	Promotes tumor invasiveness, angiogenesis, and myeloid recruitment in lung cancer, and NLRP3 inflammasome activation amplifies IL-1β/IL-18 release that can sustain protumor inflammation [69,139,140,144].
TNF-α	NF-κB activator that, when chronically expressed, fosters epithelial–mesenchymal transition, invasion, and therapy resistance across cancers, including lung [141,142,145,146].
IL-8/CXCL8	Enhances neutrophil recruitment, angiogenesis, and epithelial plasticity, and its overexpression correlates with worse outcomes in NSCLC [99,101,105].
CCL2/CCR2 and CCL5/CCR5	Chemokine circuits implicated in post-acute sequelae of coronavirus disease (PASC) are established drivers of myeloid and regulatory T-cell recruitment to tumors, favoring immunosuppression and metastasis in preclinical and clinical studies [60,61,147,148,149,150,151,152]. In LC models, interrupting CCL2–CCR2 signaling reduces tumor growth, TAM accumulation, and metastasis, supporting its role as a therapeutic target in “cold tumors” [151]. CCR5/CCL5 axis activation has been associated with advanced disease and poor outcomes across solid tumors and can augment Treg trafficking and tumor cell migration, although tumor- and context-specific effects require caution [148,149,150,153].

## Data Availability

No new data were created or analyzed in this study.

## References

[1] World Health Organization WHO Coronavirus (COVID-19) Dashboard—Global Summary of Cases and Deaths. https://data.who.int/dashboards/covid19/summary.

[2] Bray F., Laversanne M., Sung H., Ferlay J., Siegel R.L., Soerjomataram I., Jemal A. (2024). Global cancer statistics 2022: GLOBOCAN estimates of incidence and mortality worldwide for 36 cancers in 185 countries. CA Cancer J. Clin..

[3] Sung H., Ferlay J., Siegel R.L., Laversanne M., Soerjomataram I., Jemal A., Bray F. (2021). Global cancer statistics 2020: GLOBOCAN estimates of incidence and mortality worldwide for 36 cancers in 185 countries. CA Cancer J. Clin..

[4] Maxwell S.S., Weller D. (2022). Lung cancer and COVID-19: Lessons learnt from the pandemic and where do we go from here?. npj Prim. Care Respir. Med..

[5] Negoita S., Chen H., Sanchez P.V., Sherman R.L., Henley S.J., Siegel R.L., Sung H., Scott S., Benard V.B., Kohler B.A. (2024). Annual Report to the Nation on the Status of Cancer, part 2: Early assessment of the COVID-19 pandemic’s impact on cancer diagnosis. Cancer.

[6] Drescher C.W., Bograd A.J., Chang S.-C., Weerasinghe R.K., Vita A., Bell R.B. (2022). Cancer case trends following the onset of the COVID-19 pandemic: A community-based observational study with extended follow-up. Cancer.

[7] Johansson A.L.V., Larønningen S., Skovlund C.W., Kristiansen M.F., Mørch L.S., Friis S., Johannesen T.B., Myklebust T.Å., Skog A., Pettersson D. (2022). The impact of the COVID-19 pandemic on cancer diagnosis based on pathology notifications: A comparison across the Nordic countries during 2020. Int. J. Cancer.

[8] Howlader N., Bhattacharya M., Scoppa S., Miller D., Noone A.-M., Negoita S., Cronin K., Mariotto A. (2024). Cancer and COVID-19: US cancer incidence rates during the first year of the pandemic. JNCI J. Natl. Cancer Inst..

[9] Minamimoto R., Hotta M., Okafuji T., Tsutui S., Tsukuda M., Nakayama H., Shida Y., Tajima T. (2022). Change in cancer diagnosis during the COVID-19 pandemic: Trends estimated from FDG-PET/CT. Glob. Health Med..

[10] Wahidi M.M., Shojaee S., Lamb C.R., Ost D., Maldonado F., Eapen G., Caroff D.A., Stevens M.P., Ouellette D.R., Lilly C. (2020). The Use of Bronchoscopy During the Coronavirus Disease 2019 Pandemic: CHEST/AABIP Guideline and Expert Panel Report. Chest.

[11] British Thoracic Society British Thoracic Society Guidance on Respiratory Follow up of Patients with a Clinico-Radiological Diagnosis of COVID-19 Pneumonia. https://www.bsti.org.uk/media/resources/files/Resp_follow_up_guidance_post_covid_pneumonia.pdf.

[12] Rubin G.D., Ryerson C.J., Haramati L.B., Sverzellati N., Kanne J.P., Raoof S., Schluger N.W., Volpi A., Yim J.-J., Martin I.B.K. (2020). The Role of Chest Imaging in Patient Management During the COVID-19 Pandemic: A Multinational Consensus Statement from the Fleischner Society. Radiology.

[13] (2024). Surveillance, Epidemiology, and End Results (SEER) Program. Impact of COVID-19 on SEER Data Releases. National Cancer Institute. https://seer.cancer.gov/data/covid-impact.html.

[14] Howlader N., Chen H.-S., Noone A.-M., Miller D., Byrne J., Negoita S., Cronin K.A., Mariotto A.B. (2025). Impact of COVID-19 on 2021 cancer incidence rates and potential rebound from 2020 decline. JNCI J. Natl. Cancer Inst..

[15] London J.W., Fazio-Eynullayeva E., Palchuk M.B., Sankey P., McNair C. (2020). Effects of the COVID-19 pandemic on cancer-related patient encounters. JCO Clin. Cancer Inform..

[16] Societatea Română de Pneumologie (Romanian Society of Pneumology) Recomandările Societății Române de Pneumologie (SRP) şi Secţiunii de Bronhologie a SRP Privind Efectuarea Bronhoscopiei În Situaţia Actuală a Pandemiei de COVID-19 Versiunea 1/18 Martie 2020. https://srp.ro/2020/SRP_Recomand%C4%83ri%20privind%20efectuarea%20bronhoscopiei%20%C3%AEn%20situa%C5%A3ia%20actual%C4%83%20a%20pandemiei%20de%20COVID-19_ws.pdf.

[17] Kava C.M., Siegel D.A., Sabatino S.A., Qin J., Richards T.B., Jane Henley S. (2024). Lung cancer incidence, 2019–2020, United States: The potential impact of the COVID-19 pandemic. Ann. Epidemiol..

[18] Burus T., Lei F., Huang B., Christian W.J., Hull P.C., Ellis A.R., Slavova S., Tucker T.C., Lang Kuhs K.A. (2024). COVID-19 and Rates of Cancer Diagnosis in the US. JAMA Netw. Open.

[19] Bian D.J.H., Sabri S., Abdulkarim B.S. (2022). Interactions between COVID-19 and Lung Cancer: Lessons Learned during the Pandemic. Cancers.

[20] Trimarco V., Izzo R., Pacella D., Manzi M.V., Varzideh F., Lembo M., Gallo P., Piccinocchi R., Morisco C., Rozza F. (2025). The COVID-19 pandemic increased the incidence of newly diagnosed cancers: Evidence from a large cohort study in Southern Italy. BMC Med..

[21] Trojanowski M., Radomyski P., Kycler W., Michalek I.M. (2024). The impact of the COVID-19 pandemic on incidence gap in screen-detectable cancers: A cohort study in Greater Poland, Poland. Rep. Pract. Oncol. Radiother..

[22] Maringe C., Spicer J., Morris M., Purushotham A., Nolte E., Sullivan R., Rachet B., Aggarwal A. (2020). The impact of the COVID-19 pandemic on cancer deaths due to delays in diagnosis in England, UK: A national, population-based, modelling study. Lancet Oncol..

[23] Sharpless N.E. (2020). COVID-19 and cancer. Science.

[24] V’kovski P., Kratzel A., Steiner S., Stalder H., Thiel V. (2021). Coronavirus biology and replication: Implications for SARS-CoV-2. Nat. Rev. Microbiol..

[25] Malone B., Urakova N., Snijder E.J., Campbell E.A. (2022). Structures and functions of coronavirus replication-transcription complexes and their relevance for SARS-CoV-2 drug design. Nat. Rev. Mol. Cell Biol..

[26] Gordon D.E., Jang G.M., Bouhaddou M., Xu J., Obernier K., O’Meara M.J., Guo J.Z., Swaney D.L., Tummino T.A., Huettenhain R. (2020). A SARS-CoV-2 protein interaction map reveals targets for drug repurposing. Nature.

[27] Gottschalk G., Knox K., Roy A., Abid M.A., Sarosiek K. (2021). ACE2: At the Crossroad of COVID-19 and Lung Cancer. Gene Rep..

[28] Gheblawi M., Wang K., Viveiros A., Nguyen Q., Zhong J.-C., Turner A.J., Raizada M.K., Grant M.B., Oudit G.Y. (2020). Angiotensin-Converting Enzyme 2: SARS-CoV-2 Receptor and Regulator of the Renin–Angiotensin System. Circ. Res..

[29] Khiali S., Rezagholizadeh A., Entezari-Maleki T. (2022). SARS-CoV-2 and probable lung cancer risk. Bioimpacts.

[30] Jaiswal A., Shrivastav S., Kushwaha H.R., Chaturvedi R., Singh R.P. (2024). Oncogenic potential of SARS-CoV-2—Targeting hallmarks of cancer pathways. Cell Commun. Signal.

[31] Wrapp D., Wang N., Corbett K.S., Goldsmith J.A., Hsieh C.-L., Abiona O., Graham B.S., McLellan J.S. (2020). Cryo-EM structure of the 2019-nCoV spike in the prefusion conformation. Science.

[32] Lan J., Ge J., Yu J., Shan S., Zhou H., Fan S., Zhang Q., Shi X., Wang Q., Zhang L. (2020). Structure of the SARS-CoV-2 spike receptor-binding domain bound to the ACE2 receptor. Nature.

[33] Michelucci A., Sforna L., Focaia R., Leonardi M.V., Di Battista A., Rastelli G., Vespa S., Boncompagni S., Di Cristina M., Catacuzzeno L. (2025). SARS-CoV-2 ORF3a accessory protein is a water-permeable channel that induces lysosome swelling. Commun. Biol..

[34] Miller A.N., Houlihan P.R., Matamala E., Cabezas-Bratesco D., Lee G.Y., Cristofori-Armstrong B., Dilan T.L., Sanchez-Martinez S., Matthies D., Yan R. (2023). The SARS-CoV-2 accessory protein Orf3a is not an ion channel, but does interact with trafficking proteins. eLife.

[35] Sun X., Liu Y., Huang Z., Xu W., Hu W., Yi L., Liu Z., Chan H., Zeng J., Liu X. (2022). SARS-CoV-2 non-structural protein 6 triggers NLRP3-dependent pyroptosis by targeting ATP6AP1. Cell Death Differ..

[36] Merad M., Martin J.C. (2020). Pathological inflammation in patients with COVID-19: A key role for monocytes and macrophages. Nat. Rev. Immunol..

[37] Zhang Y., Chen Y., Li Y., Huang F., Luo B., Yuan Y., Xia B., Ma X., Yang T., Yu F. (2021). The ORF8 protein of SARS-CoV-2 mediates immune evasion through down-regulating MHC-I. Proc. Natl. Acad. Sci. USA.

[38] Flower T.G., Buffalo C.Z., Hooy R.M., Allaire M., Ren X., Hurley J.H. (2021). Structure of SARS-CoV-2 ORF8, a rapidly evolving immune-modulating protein. Proc. Natl. Acad. Sci. USA.

[39] Blanco-Melo D., Nilsson-Payant B.E., Liu W.C., Uhl S., Hoagland D., Møller R., Jordan T.X., Oishi K., Panis M., Sachs D. (2020). Imbalanced Host Response to SARS-CoV-2 Drives Development of COVID-19. Cell.

[40] Knoll R., Schultze J.L., Schulte-Schrepping J. (2021). Monocytes and Macrophages in COVID-19. Front. Immunol..

[41] Del Valle D.M., Kim-Schulze S., Huang H.H., Beckmann N.D., Nirenberg S., Wang B., Lavin Y., Swartz T.H., Madduri D., Stock A. (2020). An inflammatory cytokine signature predicts COVID-19 severity and survival. Nat. Med..

[42] Khalil B.A., Elemam N.M., Maghazachi A.A. (2021). Chemokines and chemokine receptors during COVID-19 infection. Comput. Struct. Biotechnol. J..

[43] Zawawi A., Naser A.Y., Alwafi H., Minshawi F. (2021). Profile of Circulatory Cytokines and Chemokines in Human Coronaviruses: A Systematic Review and Meta-Analysis. Front. Immunol..

[44] Zhang Z., Ai G., Chen L., Liu S., Gong C., Zhu X., Zhang C., Qin H., Hu J., Huang J. (2021). Associations of immunological features with COVID-19 severity: A systematic review and meta-analysis. BMC Infect. Dis..

[45] Lim S.Y., Yuzhalin A.E., Gordon-Weeks A.N., Muschel R.J. (2016). Targeting the CCL2–CCR2 signaling axis in cancer metastasis. Oncotarget.

[46] Tan Y., Wang M., Zhang Y., Ge S., Zhong F., Xia G., Sun C. (2021). Tumor-Associated Macrophages: A Potential Target for Cancer Therapy. Front. Oncol..

[47] House I.G., Savas P., Lai J., Chen A.X.Y., Oliver A.J., Teo Z.L., Todd K.L., Henderson M.A., Giuffrida L., Petley E.V. (2020). Macrophage-Derived CXCL9 and CXCL10 Are Required for Antitumor Immune Responses Following Immune Checkpoint Blockade. Clin. Cancer Res..

[48] Ayers M., Lunceford J., Nebozhyn M., Murphy E., Loboda A., Kaufman D.R., Albright A., Cheng J.D., Kang S.P., Shankaran V. (2017). IFN-γ–Related mRNA profile predicts clinical response to PD-1 blockade. J. Clin. Investig..

[49] Yu H., Lee H., Herrmann A., Buettner R., Jove R. (2014). Revisiting STAT3 signalling in cancer: New and unexpected biological functions. Nat. Rev. Cancer.

[50] Alfaro C., Sanmamed M.F., Rodríguez-Ruiz M.E., Teijeira Á., Oñate C., González Á., Ponz M., Schalper K.A., Pérez-Gracia J.L., Melero I. (2017). Interleukin-8 in cancer pathogenesis, treatment and follow-up. Cancer Treat. Rev..

[51] Ridker P.M., Everett B.M., Thuren T., MacFadyen J.G., Chang W.H., Ballantyne C., Fonseca F., Nicolau J., Koenig W., Anker S.D. (2017). Antiinflammatory Therapy with Canakinumab for Atherosclerotic Disease. N. Engl. J. Med..

[52] Mantovani A., Barajon I., Garlanda C. (2018). IL-1 and IL-1 regulatory pathways in cancer progression and therapy. Immunol. Rev..

[53] Yin K., Peluso M.J., Luo X., Thomas R., Shin M.-G., Neidleman J., Andrew A., Young K.C., Ma T., Hoh R. (2024). Long COVID manifests with T cell dysregulation, inflammation and an uncoordinated adaptive immune response to SARS-CoV-2. Nat Immunol..

[54] Phetsouphanh C., Darley D.R., Wilson D.B., Howe A., Munier C.M.L., Patel S.K., Juno J.A., Burrell L.M., Kent S.J., Dore G.J. (2022). Immunological dysfunction persists for 8 months following initial mild-to-moderate SARS-CoV-2 infection. Nat. Immunol..

[55] Su Y., Yuan D., Chen D.G., Ng R.H., Wang K., Choi J., Li S., Hong S., Zhang R., Xie J. (2022). Multiple early factors anticipate post-acute COVID-19 sequelae. Cell.

[56] Schultheiß C., Willscher E., Paschold L., Gottschick C., Klee B., Henkes S.S., Bosurgi L., Dutzmann J., Sedding D., Frese T. (2022). The IL-1β, IL-6, and TNF cytokine triad is associated with post-acute sequelae of COVID-19. Cell Rep Med..

[57] Wang K., Khoramjoo M., Srinivasan K., Gordon P.M., Mandal R., Jackson D., Sligl W., Grant M.B., Penninger J.M., Borchers C.H. (2023). Sequential multi-omics analysis identifies clinical phenotypes and predictive biomarkers for long COVID. Cell Rep. Med..

[58] Yin J.X., Agbana Y.L., Sun Z.S., Fei S.W., Zhao H.Q., Zhou X.N., Chen J.H., Kassegne K. (2023). Increased interleukin-6 is associated with long COVID-19: A systematic review and meta-analysis. Infect. Dis. Poverty.

[59] Ganesh R., Yadav S., Hurt R.T., Mueller M.R., Aakre C.A., Gilman E.A., Grach S.L., Overgaard J., Snyder M.R., Collins N.M. (2024). Pro Inflammatory Cytokines Profiles of Patients With Long COVID Differ Between Variant Epochs. J. Prim. Care Community Health.

[60] Espín E., Yang C., Shannon C.P., Assadian S., He D., Tebbutt S.J. (2023). Cellular and molecular biomarkers of long COVID: A scoping review. eBioMedicine.

[61] Bae G., Yang Z., Bucci D., Wegner C., Schäfer H., Singh Y., Lonati C., Trautwein C. (2025). Longitudinal lipoprotein and inflammatory mediators analysis uncover persisting inflammation and hyperlipidemia following SARS-CoV-2 infection in long COVID-19. Metabolomics.

[62] Ranjbar M., Rahimi A., Baghernejadan Z., Ghorbani A., Khorramdelazad H. (2022). Role of CCL2/CCR2 axis in the pathogenesis of COVID-19 and possible Treatments: All options on the Table. Int. Immunopharmacol..

[63] Sbierski-Kind J., Schlickeiser S., Feldmann S., Ober V., Grüner E., Pleimelding C., Gilberg L., Brand I., Weigl N., Ahmed M.I.M. (2024). Post COVID Care and KoCo19 study groups. Persistent immune abnormalities discriminate post-COVID syndrome from convalescence. Infection.

[64] Lai Y.J., Liu S.H., Manachevakul S., Lee T.A., Kuo C.T., Bello D. (2023). Biomarkers in long COVID-19: A systematic review. Front. Med..

[65] Çelik N., Çelik O., Laloğlu E., Özkaya A. (2023). The CXCL9/10/11-CXCR3 axis as a predictor of COVID-19 progression: A prospective, case-control study. Rev. Soc. Bras. Med. Trop..

[66] Stannard R., Lambert P.C., Lyratzopoulos G., Andersson T.M., Khan S., Rutherford M.J. (2025). The long-lasting impacts of the COVID-19 pandemic on population-based cancer survival: What are the implications for data analysis?. Br. J. Cancer.

[67] Hanna T.P., King W.D., Thibodeau S., Jalink M., Paulin G.A., Harvey-Jessop I., O’Sullivan D.E., Booth C.M. (2020). Mortality due to cancer treatment delay: Systematic review and meta-analysis. BMJ.

[68] Huang B., Lang X., Li X. (2022). The role of IL-6/JAK2/STAT3 signaling pathway in cancers. Front. Oncol..

[69] Rébé C., Ghiringhelli F. (2020). Interleukin-1β and Cancer. Cancers.

[70] Rajasegaran T., How C.W., Saud A., Ali A., Lim J.C.W. (2023). Targeting Inflammation in Non-Small Cell Lung Cancer through Drug Repurposing. Pharmaceuticals.

[71] Zhao H., Wu L., Yan G., Chen Y., Zhou M., Wu Y., Li Y. (2021). Inflammation and tumor progression: Signaling pathways and targeted intervention. Signal Transduct. Target. Ther..

[72] Skovlund C.W., Friis S., Christensen J., Nilbert M.C., Mørch L.S. (2022). Drop in cancer diagnosis during the COVID-19 pandemic in Denmark: Assessment of impact during 2020. Acta Oncol..

[73] Minamimoto R., Hotta M., Ishikane M., Inagaki T. (2020). FDG-PET/CT images of COVID-19: A comprehensive review. Glob. Health Med..

[74] Jung K.W., Won Y.J., Hong S., Kong H.J., Im J.S., Seo H.G. (2021). Prediction of Cancer Incidence and Mortality in Korea, 2021. Cancer Res. Treat..

[75] Kaufman H.W., Chen Z., Niles J., Fesko Y. (2020). Changes in the Number of US Patients With Newly Identified Cancer Before and During the Coronavirus Disease 2019 (COVID-19) Pandemic. JAMA Netw. Open.

[76] Radu G.-N., Chinezu L., Cătană R., Carabașa P., Nechifor-Boila A. (2025). The Impact of the COVID-19 Pandemic on New Lung Cancer Diagnosis in Mureș County, Romania: A 5-Year Retrospective, Comprehensive Study. Medicina.

[77] Li L., Liu Y.D., Zhan Y.T., Zhu Y.H., Li Y., Xie D., Guan X.Y. (2018). High levels of CCL2 or CCL4 in the tumor microenvironment predict unfavorable survival in lung adenocarcinoma. Thorac. Cancer.

[78] Beigel J.H., Tomashek K.M., Dodd L.E., Mehta A.K., Zingman B.S., Kalil A.C., Hohmann E., Chu H.Y., Luetkemeyer A., Kline S. (2020). Remdesivir for the Treatment of COVID-19—Final Report. N. Engl. J. Med..

[79] RECOVERY Collaborative Group (2021). Dexamethasone in Hospitalized Patients with COVID-19. N. Engl. J. Med..

[80] Gordon A.C., Mouncey P.R., Al-Beidh F., Rowan K.M., Nichol A.D., Arabi Y.M., Annane D., Beane A., van Bentum-Puijk W., REMAP-CAP Investigators (2021). Interleukin-6 Receptor Antagonists in Critically Ill Patients with COVID-19. N. Engl. J. Med..

[81] Hammond J., Leister-Tebbe H., Gardner A., Abreu P., Bao W., Wisemandle W., Baniecki M., Hendrick V., Damle B., Simón-Campos A. (2022). Oral Nirmatrelvir for High-Risk, Nonhospitalized Adults with COVID-19. N. Engl. J. Med..

[82] Herbst R.S., Morgensztern D., Boshoff C. (2018). The biology and management of non-small cell lung cancer. Nature.

[83] The Cancer Genome Atlas Research Network (2014). Comprehensive molecular profiling of lung adenocarcinoma. Nature.

[84] The Cancer Genome Atlas Research Network (2012). Comprehensive genomic characterization of squamous cell lung cancers. Nature.

[85] Alexandrov L.B., Ju Y.S., Haase K., Van Loo P., Martincorena I., Nik-Zainal S., Campbell P.J., Stratton M.R. (2016). Mutational signatures associated with tobacco smoking in human cancer. Science.

[86] Darby S., Hill D., Auvinen A., Barros-Dios J.M., Baysson H., Bochicchio F., Deo H., Falk R., Farchi S., Figueiras A. (2005). Radon in homes and risk of lung cancer: Collaborative analysis of individual data from 13 European case-control studies. BMJ.

[87] Steenland K., Mannetje A., Boffetta P., Stayner L., Attfield M., Chen J., Dosemeci M., DeKlerk N., Hnizdo E., Koskela R. (2001). Pooled exposure-response analyses and risk assessment for lung cancer in 10 cohorts of silica-exposed workers: An IARC multicentre study. Cancer Causes Control.

[88] Harris E.J.A., Musk A., de Klerk N., Reid A., Franklin P., Brims F.J.H. (2019). Diagnosis of asbestos-related lung diseases. Expert Rev. Respir. Med..

[89] Hamra G.B., Guha N., Cohen A., Laden F., Raaschou-Nielsen O., Samet J.M., Vineis P., Forastiere F., Saldiva P., Yorifuji T. (2014). Outdoor particulate matter exposure and lung cancer: A systematic review and meta-analysis. Environ. Health Perspect..

[90] Arbour K.C., Jordan E., Kim H.R., Dienstag J., Yu H.A., Sanchez-Vega F., Lito P., Berger M., Solit D.B., Hellmann M. (2018). Effects of Co-occurring Genomic Alterations on Outcomes in Patients with KRAS-Mutant Non-Small Cell Lung Cancer. Clin. Cancer Res..

[91] Ribas A., Wolchok J.D. (2018). Cancer immunotherapy using checkpoint blockade. Science.

[92] Kreuter M., Ehlers-Tenenbaum S., Palmowski K., Bruhwyler J., Oltmanns U., Muley T., Heussel C.P., Warth A., Kolb M., Herth F.J. (2016). Impact of Comorbidities on Mortality in Patients with Idiopathic Pulmonary Fibrosis. PLoS ONE.

[93] Reck M., Rodríguez-Abreu D., Robinson A.G., Hui R., Csőszi T., Fülöp A., Gottfried M., Peled N., Tafreshi A., Cuffe S. (2021). Five-Year Outcomes With Pembrolizumab Versus Chemotherapy for Metastatic Non-Small-Cell Lung Cancer With PD-L1 Tumor Proportion Score ≥ 50. J. Clin. Oncol..

[94] Hellmann M.D., Paz-Ares L., Bernabe Caro R., Zurawski B., Kim S.-W., Carcereny Costa E., Park K., Alexandru A., Lupinacci L., de la Mora Jimenez E. (2019). Nivolumab plus Ipilimumab in Advanced Non–Small-Cell Lung Cancer (CheckMate 227). N. Engl. J. Med..

[95] Herbst R.S., Giaccone G., de Marinis F., Reinmuth N., Vergnenegre A., Barrios C.H., Morise M., Felip E., Andric Z., Geater S. (2020). Atezolizumab for First-Line Treatment of PD-L1-Selected Patients with NSCLC. N. Engl. J. Med..

[96] Sezer A., Kilickap S., Gümüş M., Bondarenko I., Özgüroğlu M., Gogishvili M., Turk H.M., Cicin I., Bentsion D., Gladkov O. (2021). Cemiplimab monotherapy for first-line treatment of advanced non-small-cell lung cancer with PD-L1 of at least 50%: A multicentre, open-label, global, phase 3, randomised, controlled trial. Lancet.

[97] Özgüroğlu M., Sezer A., Kilickap S., Gümüş M., Bondarenko I., Gogishvili M., Nechaeva M., Schenker M., Cicin I., Ho G.F. (2023). First-line cemiplimab monotherapy and continued cemiplimab beyond progression plus chemotherapy for advanced non-small-cell lung cancer with PD-L1 50% or more (EMPOWER-Lung 1): 35-month follow-up from a mutlicentre, open-label, randomised, phase 3 trial. Lancet Oncol..

[98] Zhang N., Zeng Y., Du W., Zhu J., Shen D., Liu Z., Huang J.A. (2016). The EGFR pathway is involved in the regulation of PD-L1 expression via the IL-6/JAK/STAT3 signaling pathway in EGFR-mutated non-small cell lung cancer. Int. J. Oncol..

[99] Kuo I.Y., Yang Y.E., Yang P.S., Tsai Y.J., Tzeng H.T., Cheng H.C., Kuo W.T., Su W.C., Chang C.P., Wang Y.C. (2021). Converged Rab37/IL-6 trafficking and STAT3/PD-1 transcription axes elicit an immunosuppressive lung tumor microenvironment. Theranostics.

[100] Liu H., Zhao Q., Tan L., Wu X., Huang R., Zuo Y., Chen L., Yang J., Zhang Z.X., Ruan W. (2023). Neutralizing IL-8 potentiates immune checkpoint blockade efficacy for glioma. Cancer Cell.

[101] Zou D., Song A., Yong W. (2023). Prognostic role of IL-8 in cancer patients treated with immune checkpoint inhibitors: A system review and meta-analysis. Front. Oncol..

[102] Rizzo M., Varnier L., Pezzicoli G., Pirovano M., Cosmai L., Porta C. (2022). IL-8 and its role as a potential biomarker of resistance to anti-angiogenic agents and immune checkpoint inhibitors in metastatic renal cell carcinoma. Front. Oncol..

[103] Zhai X., Zhang J., Tian Y., Li J., Jing W., Guo H., Zhu H. (2020). The mechanism and risk factors for immune checkpoint inhibitor pneumonitis in non-small cell lung cancer patients. Cancer Biol. Med..

[104] O’Leary C.L., Pierce N., Patel S.P., Naidoo J. (2024). Immune-Related Toxicity in NSCLC: Current State-of-the-Art and Emerging Clinical Challenges. J. Thorac. Oncol..

[105] Lin M.X., Zang D., Liu C.G., Han X., Chen J. (2024). Immune checkpoint inhibitor-related pneumonitis: Research advances in prediction and management. Front. Immunol..

[106] Xu Y., Chen R., Pan R., Gao X., Huang H., Wang M. (2025). Clinical management of checkpoint inhibitor pneumonitis: Focus, challenges, and future directions. Chin. Med. J. Pulm. Crit. Care Med..

[107] Nishino M. (2022). Imaging of Oncologic Treatment-Related Pneumonitis: A Focused Review on Emerging Issues of Immune Checkpoint Inhibitor Pneumonitis, From the *AJR* Special Series on Inflammation. AJR Am. J. Roentgenol..

[108] Picasso R., Cozzi A., Picasso V., Zaottini F., Pistoia F., Perissi S., Martinoli C. (2023). Immune checkpoint inhibitor-related pneumonitis and COVID-19: A case-matched comparison of CT findings. Radiol. Med..

[109] Hao Y., Zhang X., Yu L. (2022). Immune checkpoint inhibitor-related pneumonitis in non-small cell lung cancer: A review. Front. Oncol..

[110] Guo M., Liu J., Miao R., Ahmed Z., Yu J., Guan J., Ahmad S., Zhou S., Grove A., Manoucheri M. (2022). A Single Center Retrospective Study of the Impact of COVID-19 Infection on Immune-related Adverse Events in Cancer Patients Receiving Immune Checkpoint Inhibitors. J. Immunother..

[111] Mandala M., Lorigan P., De Luca M., Bianchetti A., Merelli B., Bettini A.C., Bonomi L., Nahm S., Vitale M.G., Negrini G. (2021). SARS-CoV-2 infection and adverse events in patients with cancer receiving immune checkpoint inhibitors: An observational prospective study. J. Immunother. Cancer.

[112] Ackermann M., Verleden S.E., Kuehnel M., Haverich A., Welte T., Laenger F., Vanstapel A., Werlein C., Stark H., Tzankov A. (2020). Pulmonary Vascular Endothelialitis, Thrombosis, and Angiogenesis in COVID-19. N. Engl. J. Med..

[113] Afzali B., Noris M., Lambrecht B.N., Kemper C. (2022). The State of Complement in COVID-19. Nat. Rev. Immunol..

[114] Chauhan A.J., Wiffen L.J., Brown T.P. (2020). COVID-19: A collision of complement, coagulation and inflammatory pathways. J. Thromb. Haemost..

[115] Zuo Y., Yalavarthi S., Shi H., Gockman K., Zuo M., Madison J.A., Blair C.N., Weber A., Barnes B.J., Egeblad M. (2020). Neutrophil extracellular traps in COVID-19. JCI Insight.

[116] Middleton E.A., He X.-Y., Denorme F., Campbell R.A., Ng D., Salvatore S.P., Mostyka M., Baxter-Stoltzfus A., Borczuk A.C., Loda M. (2020). Neutrophil extracellular traps contribute to immunothrombosis in COVID-19 acute respiratory distress syndrome. Blood.

[117] Zhu Y., Chen X., Liu X. (2022). NETosis and Neutrophil Extracellular Traps in COVID-19: Immunothrombosis and Beyond. Front. Immunol..

[118] Magro C., Mulvey J.J., Berlin D., Nuovo G., Salvatore S., Harp J., Baxter-Stoltzfus A., Laurence J. (2020). Complement associated microvascular injury and thrombosis in the pathogenesis of severe COVID-19 infection: A report of five cases. Transl. Res..

[119] Gianni P., Goldin M., Ngu S., Zafeiropoulos S., Geropoulos G., Giannis D. (2022). Complement-mediated microvascular injury and thrombosis in the pathogenesis of severe COVID-19: A review. World J. Exp. Med..

[120] Lee M.H., Perl D.P., Steiner J., Pasternack N., Li W., Maric D., Safavi F., Horkayne-Szakaly I., Jones R., Stram M.N. (2022). Neurovascular injury with complement activation and inflammation in COVID-19. Brain.

[121] Low R.N., Low R.J., Akrami A. (2023). A review of cytokine-based pathophysiology of Long COVID symptoms. Front. Med..

[122] Queiroz M.A.F., das Neves P.F.M., Lima S.S., Lopes J.D.C., Torres M.K.D.S., Vallinoto I.M.V.C., Bichara C.D.A., dos Santos E.F., de Brito M.T.F.M., da Silva A.L.S. (2022). Cytokine Profiles Associated With Acute COVID-19 and Long COVID-19 Syndrome. Front. Cell. Infect. Microbiol..

[123] Bédard-Matteau J., Soulé A., Liu K.Y., Fourcade L., Fraser D.D., Emad A., Rousseau S. (2024). Circulating IL-17F, but not IL-17A, is elevated in severe COVID-19 and leads to an ERK1/2 and p38 MAPK-dependent increase in ICAM-1 cell surface expression and neutrophil adhesion on endothelial cells. Front. Immunol..

[124] Sharif-Askari F.S., Sharif-Askari N.S., Hafezi S., Mdkhana B., Alsayed H.A.H., Ansari A.W., Mahboub B., Zakeri A.M., Temsah M.H., Zahir W. (2022). Interleukin-17, a salivary biomarker for COVID-19 severity. PLoS ONE.

[125] Patterson B.K., Guevara-Coto J., Mora J., Francisco E.B., Yogendra R., Mora-Rodríguez R.A., Beaty C., Lemaster G., Do G.K., Katz A. (2024). Long COVID diagnostic with differentiation from chronic lyme disease using machine learning and cytokine hubs. Sci. Rep..

[126] Ogarek N., Oboza P., Olszanecka-Glinianowicz M., Kocelak P. (2023). SARS-CoV-2 infection as a potential risk factor for the development of cancer. Front. Mol. Biosci..

[127] Rudroff T. (2025). Convergent Mechanisms in Virus-Induced Cancers: A Perspective on Classical Viruses, SARS-CoV-2, and AI-Driven Solutions. Infect. Dis. Rep..

[128] Cho J.L., Villacreses R., Nagpal P., Guo J., Pezzulo A.A., Thurman A.L., Hamzeh N.Y., Blount R.J., Fortis S., Hoffman E.A. (2022). Quantitative Chest CT Assessment of Small Airways Disease in Post-Acute SARS-CoV-2 Infection. Radiology.

[129] Grist J.T., Collier G.J., Walters H., Kim M., Chen M., Abu Eid G., Laws A., Matthews V., Jacob K., Cross S. (2022). Lung Abnormalities Detected with Hyperpolarized 129Xe MRI in Patients with Long COVID. Radiology.

[130] Hirano T. (2021). IL-6 in inflammation, autoimmunity and cancer. Int. Immunol..

[131] Brown S.W., Dobelle M., Padilla M., Agovino M., Wisnivesky J.P., Hashim D., Boffetta P. (2019). Idiopathic Pulmonary Fibrosis and Lung Cancer. A Systematic Review and Meta-analysis. Ann. Am. Thorac. Soc..

[132] Zhang L., Richards A., Barrasa M.I., Hughes S.H., Young R.A., Jaenisch R. (2021). Reverse-transcribed SARS-CoV-2 RNA can integrate into the genome of cultured human cells and can be expressed in patient-derived tissues. Proc. Natl. Acad. Sci. USA.

[133] Parry R., Gifford R.J., Lytras S., Ray S.C., Coin L.J.M. (2021). No evidence of SARS-CoV-2 reverse transcription and integration as the origin of chimeric transcripts in patient tissues. Proc. Natl. Acad. Sci. USA.

[134] Briggs E., Ward W., Rey S., Law D., Nelson K., Bois M., Ostrov N., Lee H.H., Laurent J.M., Mita P. (2021). Assessment of potential SARS-CoV-2 virus integration into human genome reveals no significant impact on RT-qPCR COVID-19 testing. Proc. Natl. Acad. Sci. USA.

[135] Chen Y.S., Lu S., Zhang B., Du T., Li W.J., Lei M., Zhou Y., Zhang Y., Liu P., Sun Y.Q. (2022). Comprehensive analysis of RNA-seq and whole genome sequencing data reveals no evidence for SARS-CoV-2 integrating into host genome. Protein Cell.

[136] Smits N., Rasmussen J., Bodea G.O., Amarilla A.A., Gerdes P., Sanchez-Luque F.J., Ajjikuttira P., Modhiran N., Liang B., Faivre J. (2021). No evidence of human genome integration of SARS-CoV-2 found by long-read DNA sequencing. Cell Rep..

[137] Park M.H., Hong J.T. (2016). Roles of NF-κB in Cancer and Inflammatory Diseases and Their Therapeutic Approaches. Cells.

[138] Ben-Baruch A. (2022). Tumor Necrosis Factor α: Taking a Personalized Road in Cancer Therapy. Front. Immunol..

[139] Shadab A., Mahjoor M., Abbasi-Kolli M., Afkhami H., Moeinian P., Safdarian A.-R. (2023). Divergent functions of NLRP3 inflammasomes in cancer: A review. Cell Commun. Signal..

[140] Guo H., Callaway J.B., Ting J.P. (2015). Inflammasomes: Mechanism of action, role in disease, and therapeutics. Nat. Med..

[141] Wu Y., Zhou B. (2010). TNF-*α*/NF-*κ*B/Snail pathway in cancer cell migration and invasion. Br. J. Cancer.

[142] Tang D., Tao D., Fang Y., Deng C., Xu Q., Zhou J. (2017). TNF-Alpha Promotes Invasion and Metastasis via NF-Kappa B Pathway in Oral Squamous Cell Carcinoma. Med. Sci. Monit. Basic Res..

[143] Caetano M.S., Zhang H., Cumpian A.M., Gong L., Unver N., Ostrin E.J., Daliri S., Chang S.H., Ochoa C.E., Hanash S. (2016). IL6 Blockade Reprograms the Lung Tumor Microenvironment to Limit the Development and Progression of K-ras-Mutant Lung Cancer. Cancer Res..

[144] Pretre V., Papadopoulos D., Regard J., Pelletier M., Woo J. (2022). Interleukin-1 (IL-1) and the inflammasome in cancer. Cytokine.

[145] Ma Q., Hao S., Hong W., Tergaonkar V., Sethi G., Tian Y., Duan C. (2024). Versatile function of NF-ĸB in inflammation and cancer. Exp. Hematol. Oncol..

[146] Capece D., Verzella D., Flati I., Arboretto P., Cornice J., Franzoso G. (2022). NF-κB: Blending metabolism, immunity, and inflammation. Trends Immunol..

[147] Fei L., Ren X., Yu H., Zhan Y. (2021). Targeting the CCL2/CCR2 Axis in Cancer Immunotherapy: One Stone, Three Birds?. Front. Immunol..

[148] González-Arriagada W.A., Coletta R.D., Lozano-Burgos C., García C., Maripillán J., Alcayaga-Miranda F., Godínez-Pacheco B., Oyarce-Pezoa S., Martínez-Flores R., García I.E. (2023). CR5/CCL5 axis is linked to a poor outcome, and inhibition reduces metastasis in oral squamous cell carcinoma. J. Cancer Res. Clin. Oncol..

[149] Zhang X.F., Zhang X.L., Wang Y.J., Fang Y., Li M.L., Liu X.Y., Luo H.Y., Tian Y. (2023). The regulatory network of the chemokine CCL5 in colorectal cancer. Ann. Med..

[150] Shan J., Xu Y., Lun Y. (2024). Comprehensive analysis of the potential biological significance of CCL5 in pan-cancer prognosis and immunotherapy. Sci. Rep..

[151] Sedighzadeh S.S., Khoshbin A.P., Razi S., Keshavarz-Fathi M., Rezaei N. (2021). A narrative review of tumor-associated macrophages in lung cancer: Regulation of macrophage polarization and therapeutic implications. Transl. Lung Cancer Res..

[152] Liner A.G., van Gogh M., Roblek M., Heikenwalder M., Borsig L. (2025). Non-redundant roles of the CCR1 and CCR2 chemokine axes in monocyte recruitment during lung metastasis. Neoplasia.

[153] Singh S.K., Mishra M.K., Eltoum I.E.A., Bae S., Lillard J.W., Singh R. (2018). CCR5/CCL5 axis interaction promotes migratory and invasiveness of pancreatic cancer cells. Sci. Rep..

[154] Montani D., Savale L., Noel N., Meyrignac O., Colle R., Gasnier M., Corruble E., Beurnier A., Jutant E.M., Pham T. (2022). Post-acute COVID-19 syndrome. Eur. Respir. Rev..

[155] Miao G., Zhao H., Li Y., Ji M., Chen Y., Shi Y., Bi Y., Wang P., Zhang H. (2021). ORF3a of the COVID-19 virus SARS-CoV-2 blocks HOPS complex-mediated assembly of the SNARE complex required for autolysosome formation. Dev. Cell.

[156] Zhang Y., Sun H., Pei R., Mao B., Zhao Z., Li H., Lin Y., Lu K. (2021). The SARS-CoV-2 protein ORF3a inhibits fusion of autophagosomes with lysosomes. Cell Discov..

[157] JafariNezhad A.R., YektaKooshali M.H. (2018). Lung cancer in idiopathic pulmonary fibrosis: A systematic review and meta-analysis. PLoS ONE.

[158] Thuya W.L., Cao Y., Ho P.C.-L., Wong A.L.-A., Wang L., Zhou J., Nicot C., Goh B.C. (2025). Insights into IL-6/JAK/STAT3 signaling in the tumor microenvironment: Implications for cancer therapy. Cytokine Growth Factor Rev..

[159] Kim T.S., Shin E.C. (2019). The activation of bystander CD8^+^ T cells and their roles in viral infection. Exp. Mol. Med..

[160] Cantini L., Mentrasti G., Russo G.L., Signorelli D., Pasello G., Rijavec E., Russano M., Antonuzzo L., Rocco D., Giusti R. (2022). Evaluation of COVID-19 impact on DELAYing diagnostic-therapeutic pathways of lung cancer patients in Italy (COVID-DELAY study): Fewer cases and higher stages from a real-world scenario. ESMO Open.

